# Physiological Responses and Ovarian Development of Female Chinese Mitten Crab *Eriocheir sinensis* Subjected to Different Salinity Conditions

**DOI:** 10.3389/fphys.2017.01072

**Published:** 2018-01-04

**Authors:** Xiaowen Long, Xugan Wu, Lei Zhao, Haihui Ye, Yongxu Cheng, Chaoshu Zeng

**Affiliations:** ^1^Department of Marine Biology and Technology, College of Ocean and Earth Sciences, Xiamen University, Xiamen, China; ^2^Centre for Research on Environmental Ecology and Fish Nutrition of Ministry of Agriculture, Shanghai Ocean University, Shanghai, China; ^3^Shanghai Engineering Research Center of Aquaculture, Shanghai Ocean University, Shanghai, China; ^4^National Demonstration Centre for Experimental Fisheries Science Education, Shanghai Ocean University, Shanghai, China; ^5^Centre for Sustainable Tropical Fisheries and Aquaculture, College of Marine & Environmental Sciences, James Cook University, Townsville, QLD, Australia

**Keywords:** *Eriocheir sinensis*, females, salinity, ovarian development, physiological responses

## Abstract

Salinity plays a key role affecting ovarian development, osmoregulation and metabolism of female Chinese mitten crab, *Eriocheir sinensis* during reproductive migration. In this study, female *E. sinensis* after their puberty molt were subjected to four salinities of 0, 6, 12, and 18‰ for 40 days to investigate the salinity effects on their ovarian development as well as a range of important physiological parameters. Elevated salinity accelerated the ovarian development with ovigerous crabs found at salinity treatments of 12 and 18‰ despite no copulation had occurred. Meanwhile the survival rate of female crabs showed a decreasing trend with increasing salinity. Higher salinity also led to increased hemolymph Na^+^, K^+^, Ca^2+^, Cl^−^, and Mg^2+^ concentrations. The 6‰ treatment had the highest contents of hemolymph total and major free amino acids while the Na^+^/K^+^ -ATPase activity in the posterior gills was the lowest among treatments. Total n-3 polyunsaturated fatty acids (∑n-3PUFA) and n-3/n-6 PUFA ratio in the anterior gills showed a decreasing trend with salinity while 18‰ had the highest ∑PUFA and ∑n-6PUFA. The ∑n-3PUFA content and n-3/n-6 PUFA ratio of the posterior gills showed a fluctuating pattern and the highest value was detected at 0‰, while an increasing trend was found for the ∑n-6PUFA with increasing salinity. The hemolymph glucose showed a decreasing trend with increasing salinity and the highest total cholesterol in hemolymph was detected at 12‰. The 18‰ treatment had the highest levels of hemolymph γ-glutamyltransferase, alkaline phosphatase and acid phosphatase, as well as glucose, urea and acid phosphatase in hepatopancreas while the highest hemolymph superoxide dismutase and malondialdehyde were detected at 0‰. Overall, the results showed that salinity increase from freshwater to brackish conditions led to lower metabolism, accelerated ovarian development, and the appearance of ovigerous crabs without copulation in female *E. sinensis* post puberty molt.

## Introduction

The Chinese mitten crab, *Eriocheir sinensis*, earned its name from the distinguishing feature of dense patches of hairs on its chelipeds. The freshwater crab species is native to China and is distributed widely in streams and rivers along the eastern coasts of China (Sui et al., 2009; Zeng et al., 2012). The species has also been introduced to Europe and America and has become established locally, which is considered as an invasive species (Anger, 1991; ref for American case). The Chinese mitten crab spends most of its live in freshwater, but after puberty molt, sexual mature crabs migrate downstream to estuaries for reproduction (Zhang et al., 2001; Cheng et al., 2008). During the reproductive migration, the gonads of the crabs gradually develop and finally reach mature after arriving in the estuaries where they copulate and spawn (Zhang, 1973; Bentley, 2011). Salinity is hence considered a key environmental factor that affects gonadal development, reproduction and physiological status of sexual mature *E. sinensis*. It is well-known salinity in estuaries fluctuates substantially due to tides and freshwater runoff from rivers and streams (Wang and Xu, 2003); indeed, salinity of Yangtze River estuaries, where *E. sinensis* migrate to for reproduction, reportedly fluctuates between 3.4 and 17‰ (Zhang, 1973; Zhang and Li, 2002). Due to changes in salinity condition during the reproductive migration, as *E. sinensis* migrating toward estuaries, osmoregulatory mechanisms are expected to be mobilized to cope with such change (Wang and Xu, 2003; Jia et al., 2012).

*E. sinensis* is known as a euryhaline species and a strong osmoregulator (Rathmayer and Siebers, 2001; Wang et al., 2012). It is well-documented that various ions and free amino acids are the main contributors to hemolymph osmolality of crustaceans; as facilitated by various ion transport enzymes and proteins, they play crucial roles in crustacean osmoregulation (Towle and Weihrauch, 2001; Romano and Zeng, 2012). Among the ion transportation enzymes and proteins, Na^+^/K^+^-ATPase in the posterior gills of crustaceans is well-known highly important (Genovese et al., 2004; Cieluch et al., 2007).

Past studies on effects of salinity on *E. sinensis* have mostly focused on either a single aspect of physiological change and/or over short-term exposure (Wang and Xu, 2003; Lu et al., 2011; Jia et al., 2012). To date, there is a lack of comprehensive study on osmoregulation and physiological responses of *E. sinensis* exposed to different salinity conditions, particularly in the case of long-term exposure. Moreover, while it has been shown that brackish water (15‰) promoted gonadal development of *E. sinensis* following puberty molting (Wu et al., 2013), it is unclear whether and how effects of salinity on gonadal development of *E. sinensis* are correlated with other important physiological processes, such as osmoregulation and metabolism.

Previous studies have also shown clear gender differences in the onset of reproductive migration of *E. sinensis*, for example, the arriving time in estuaries is different between males and females. The female crabs generally start reproductive migration earlier and arrive in estuaries first where they wait for males to arrive during the time their ovaries further mature; once males arrive in estuaries, copulation occurs before they depart again and the females move further downstream to spawn and hatch larvae (Lai, 1994; Zhang and Li, 2002; Bentley, 2011). As such, there are likely gender differences in physiological responses to salinity changes during reproductive migration and gonadal development, which necessitates studies on salinity effects on gonadal development of *E. sinensis* being carried out separately for males and females. Our recent studies have reported the effects of long-term salinity adaptation on gonadal development, osmoregulation and metabolism of adult male *E. sinensis* (Long et al., 2017b), however it remains unknown the physiological responses and ovarian development of female *E. sinensis* subjected to different salinity conditions. Therefore, the present study comprehensively investigated the effects of long-term exposure to different salinities on ovarian development, hemolymph osmolality and major ion as well as free amino acid concentrations, gill Na^+^/K^+^-ATPase activity and fatty acids profile, as well as a range of metabolic and antioxidant indices in hemolymph and hepatopancreas of the female *E. sinensis* following puberty molt.

## Materials and methods

### Experimental design and set up

Female *E. sinensis* used for the experiments were obtained from a crab farm in Yangchenghu Lake, Jiangsu province, China in early October, 2013 following their puberty molt. They were transported to Fengxian aquaculture research center, Shanghai Fisheries Research Institute, Shanghai, China, where the salinity experiment was conducted. Only active and appendage intact females were selected and randomly stocked into 8 indoor polyethylene tanks (length × width × depth = 2.5 × 3.75 × 1.0 m) for the experiment. To confirm gonadal development status of the crabs, at the beginning of the experiment, 15 females were randomly selected and dissected to obtain ovaries. The ovaries from each crab were subsequently weighed to calculate gonadosomatic index (GSI). With the mean GSI of the sampled crabs at only 3.78 ± 0.64%, the results confirmed that these crabs had similar immature gonads. The initial body weight (BW) of the female crabs ranged from 100 to 120 g. Approximately 40% bottom area of the tanks was covered by 10–20 cm fine sand and pieces of polyvinyl chloride (PVC) tubes (diameter: 15 cm) and tiles were provided as shelters for crabs. Four salinity treatments, 0, 6, 12, and 18‰, were set up, and each treatment had two replicate tanks with 30 crabs stocked in a tank. During the experiment, water depth of each tank was maintained at 70 cm. The initially salinity in all tanks was 0‰, following the stocking of the crabs, salinity in the tanks allocated for higher salinity treatments was gradually increased to the designated levels, respectively at a rate of 3‰ day ^−1^ by adding brine.

The experiment started on 11th October, 2013 and lasted for 40 days. During the experiment, the crabs were fed daily at 18: 00 with trash fish and residue of the feed was removed next morning. The feeding ration was adjusted based on water temperature and leftover food during the experiment, i.e., 3–5% total biomass when water temperature >20°C but reduced to 1–3% of total biomass at 15–20°C. Throughout the experiment, all rearing tanks were aerated and photoperiod was set at 12 h light: 12 h dark with fluorescent lamps (40 W) as the lighting source. The water temperature in each tank was measured daily at 12:00 and 22:00, and ammonia-N, nitrite, dissolved oxygen (DO) and pH were measured every 3 days. Water in each tank was exchanged based on water parameter readings in order to keep water quality parameters within following ranges: ammonia-N < 0.5 mg L^−1^; nitrite < 0.15 mg L^−1^; DO > 4 mg L^−1^ and pH 7.0–8.5 throughout the experiment.

### Sampling procedure

All crabs were starved for 24 h prior to sampling on day 40. Four crabs were randomly sampled from each tank and their weights measured using a digital balance (precision: 0.01 g). All crabs were treated with cold shock method to minimize suffering, then around 2 mL hemolymph was withdrawn from each crab sample by inserting a 1.0 mL syringe at the base of the third walking leg and all hemolymph samples were stored at −40°C for later analysis. It is known that *E. sinensis* has six pairs of gills, previous studies have shown that the first 3 pairs of the gills (pairs 1–3, also known as anterior gills) are responsible for respiration while pairs 4–6 of gills (posterior gills) are specified for osmoregulation (Long et al., 2017b). Therefore, in this study, the pairs 5 posterior gills were sampled and snap frozen in liquid nitrogen and stored at −80°C for the Na^+^/K^+^-ATPase activity and its mRNA expression analysis. The remaining anterior and posterior gills were stored at −40°C for later fatty acid analysis. The ovary and hepatopancrea of each crab were also dissected and their weight measured before being stored at −40°C for various biochemical analysis.

Based on the ovarian and hepatopancreas weights obtained, the gonadosomatic index (GSI) and hepatosomatic index (HSI) of each crab were calculated using following formulas:

$$\begin{array}{l} GSI=\frac{M_{G}}{M_{C}}\times 100\%\\ HSI=\frac{M_{H}}{M_{C}}\times 100\% \end{array}$$

where *M*_*G*_ is gonad weight, *M*_*H*_ is hepatopancreas weight and *M*_*C*_ is crab weight.

### Hemolymph osmolality and ionic concentrations

The hemolymph samples were firstly thawed and homogenized using an IKA homogenizer (T10B, IKA Co., Germany). The resultant homogenates were then centrifuged at 10,000 × g for 20 min at 4°C, and the supernatant was collected for analysis. The hemolymph osmolality was analyzed using a freezing-point osmometer (OSMOMAT 030, Gonotec, Berlin, Germany). The concentrations of Na^+^, K^+^, Ca^2+^, and Cl^−^ were detected using an electrolyte analyzer (K-Lite 5, Meizhou Kangli high-tech Co., Ltd, Guangzhou, China), while the concentration of Mg^2+^ was determined with a spectrophotometer (T6 New Century, Beijing Purkinje General Instrument Co., Ltd, Beijing, China) and a commercial test kit (Nanjing Jiancheng Bioengineering Institute, Nanjing, China).

### Free amino acids in hemolymph

Each hemolymph sample was de-proteinized by adding an equal volume of 12% (m: v) trichloroacetic acid (TCA) (Chew et al., 1999) and subsequently vibrated using SCILOGEX MX-S vortex mixer (SCILOGEX, LLC, Berlin, CT, USA) before being placed in 4°C freezer for 20 min. The mixture was then centrifuged at 10,000 × g and 4°C for 10 min using an Eppendorf centrifuge (5417R, Eppendorf, Hamburg, Germany). The supernatant was collected and pH adjusted to 2.2 by adding 6 M NaOH solution. The analysis of free amino acids (FAAs) was performed with a Hitachi L-8900 Amino Acid Analyzer (Hitachi, Ltd., Tokyo, Japan) with a Li column (Inter diameter × length = 4.6 × 60 mm), which packed with Hitachi custom ion exchange resin #2622 (Particle size: 3 μm).

### Gill Na^+^/K^+^-ATPase activity and mRNA expression

Approximately 0.2 g posterior gill from each crab was homogenized in 1 mL ice-cold homogenization buffer [1 mol L^−1^ Tris-HCl (pH = 7.6): 10 mL; NaCl: 2.925 g; EDTA: 0.05 g; 100 mmol L^−1^ PMSF: 0.5 mL; and dilute to 500 mL with distilled water] using IKA homogenizer (T10B, IKA Co., Germany). The homogenate was centrifuged at 10,000 × g for 10 min at 4°C, and the supernatant was collected for subsequent analysis (Long et al., 2017b). The Na^+^/K^+^ -ATPase activity and total soluble protein in the supernatant were determined with a spectrophotometer (T6 New Century, Beijing Purkinje General Instrument Co., Ltd, Beijing, China) at 540 and 595 nm absorbance, respectively, and the assays were performed using respective commercial assay kits (Nanjing Jiancheng Bioengineering Institute, Jiangsu, China) according to manufacture's instruction.

For measuring mRNA expression level of Na^+^/K^+^-ATPase, the frozen posterior gills were grounded in a mortar with liquid nitrogen, and then total RNA was isolated using a RNA extracting kit (Cat. D9108A, Takara Biotechology Co., Ltd., Dalian, China) following the manufacture's protocol. The final total RNA was dissolved in 200 mL RNase-free water. The concentration of total RNA was determined using a Nano-Drop 2000 spectrophotometer (Thermo, Scientific, USA) and the RNA integrity was checked with 1% agarose gel electrophoresis. One hundred ng of total RNA was subsequently used as reverse-transcription template for the synthesis of first strand cDNA using a reverse transcription kit (Cat.D2639A, Takara Biotechology Co., Ltd., Dalian, China). According to Na^+^/K^+^-ATPase gene sequence of *E. sinensis*, specific primers were designed by Primer Premier 5.0 software, and the sequence was shown in Table 1. The β-actin gene of *E. sinensis* was used as a reference gene.

**Table 1:** Primers for quantitative real-time PCR of Na^+^/K^+^-ATPase α1 gene of female *E. sinensis*.

**Primer**	**Sequence (5′-3′)**	**NCBI accession number or reference**
NAK-RT F	TGAATGACTCCCCAGCTCTCAAGA	AF3011581.1
NAK-RT R	CAGAATCATGTCAGCAGCCTGCTT	
*β-actin* 1 F	TCATCACCATCGGCAATGA	Guo et al., 2013
*β-actin* 1 R	TTGTAAGTGGTCTCGTGGATG	HM053699.1

*Note that this table cited in the previously published article by Long et al. (2017b)*.

### Gill fatty acid profile

The anterior and posterior gills of crabs from each experimental tank were pooled, homogenized and freeze-dried, respectively. Total lipids in the gill samples were extracted based on the method of (Folch et al., 1957) Fatty acid methyl esters (FAMEs) were prepared by boiling 14% boron trifluoride/methanol (w/w) (Morrison and Smith, 1964). FAMEs were analyzed by flame ionization detection (FID) after injecting a sample into a Thermo Trace GC Ultra gas chromatograph fitted with a 100 × 0.25 mm ID (0.2 μm film thickness) Supelco SP-2560 capillary column (Supelco, Inc., Billefonte, PA, USA). Injector and detector temperatures were 260°C. The column temperature was initially held at 70°C, followed by an increase at a rate of 50°C min ^−1^ to 140°C and held for 1 min, then increase to 180°C at 4°C min ^−1^ and held for 1 min. It was then further increased at 3°C min ^−1^ to the final temperature of 225°C and held for 30 min until all FAMEs had been eluted. The carrier gas was nitrogen with the flow velocity at 1 mL min ^−1^. Peaks were identified by comparing retention times with known standard (Sigma-Aldrich Co., St. Louis, MO, USA). Fatty acid profile was expressed as percentage of each fatty acid to the total fatty acids (% total fatty acids) based on the area percentage.

### Other biochemical parameters in hemolymph and hepatopancreas

Approximately 0.2 g hepatopancreas tissue from each crab was homogenized in 1 mL icy physiological saline [210 mmol L^−1^ NaCl; 13.6 mmol L^−1^ KCl; 3.8 mmol L^−1^ MgCl_2_; 2.6 mmol L^−1^ Na_2_SO_4_; 10 mmol L^−1^ Hepse (pH 7.5)] with an IKA homogenizer (T10B, IKA Co., Germany). The homogenate was centrifuged at 10,000 × g for 20 min at 4°C and the supernatant was collected for subsequent analysis. Similarly, the hemolymph samples were firstly thawed, subsequently homogenized and centrifuged by the same procedure, and the supernatant was collected for later biochemical analysis.

The total cholesterol, triglyceride, high-density lipoprotein cholesterol, low-density lipoprotein cholesterol, uric acid, urea, glucose, alkaline phosphatase, and γ-glutamyltranspeptidase were analyzed using an automatic biochemical analyzer (BS-200, Shenzhen Mindray Bio-Medical Electronics Co., Ltd., China) and commercial bio-kits (Shanghai Jinxi Biotech Co., Ltd, Shanghai, China). The activity of superoxide dismutase and acid phosphatase was detected according to (Qiu et al., 2011) and Feng et al. (2013), while the malonaldehyde was determined by the thiobarbituric acid method (Ohkawa et al., 1979) using a commercial kit (Nanjing Jiancheng Bioengineering Institute, Jiangsu, China).

### Statistical analysis

Data are presented as mean ± standard error (SE). Homogeneity of variance was tested with Levene's test. When necessary, arcsine-square root of logarithmic transformation was performed prior to analysis. Statistical analyses were conducted using one-way ANOVA and compared with Duncan's multiple range test. *P* < 0.05 was regard as statistically significant difference. All statistical analysis was performed using SPSS package (version 16.0).

## Results

### Survival, GSI, and HSI

**Figure 1:**
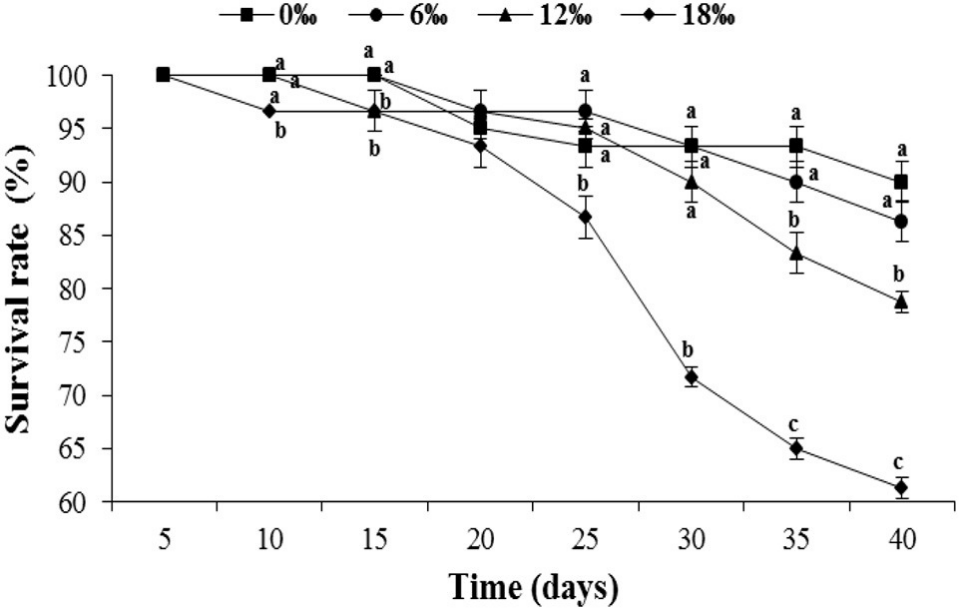
The survival rate (%) (± SE) of the female *Eriocheir sinensis* subjected to different salinities for 40 days. Different letters on the top of different tag types in the same column indicated significant differences.

The survival rates of the crabs in all four salinity treatments initially remained high and similar during the first 20 days but showed a more substantial decline from day 25 onward, particularly for the highest salinity treatment (18‰). By the end of the experiment (day 40), crab survival showed a trend of decrease with increasing salinity, ranged from 61.3% of the 18 ‰ treatment to 90.0% of the 0‰ treatment (Figure 1). The differences of the final survival among treatments are statistically significant (*P* < 0.05).

**Figure 2:**
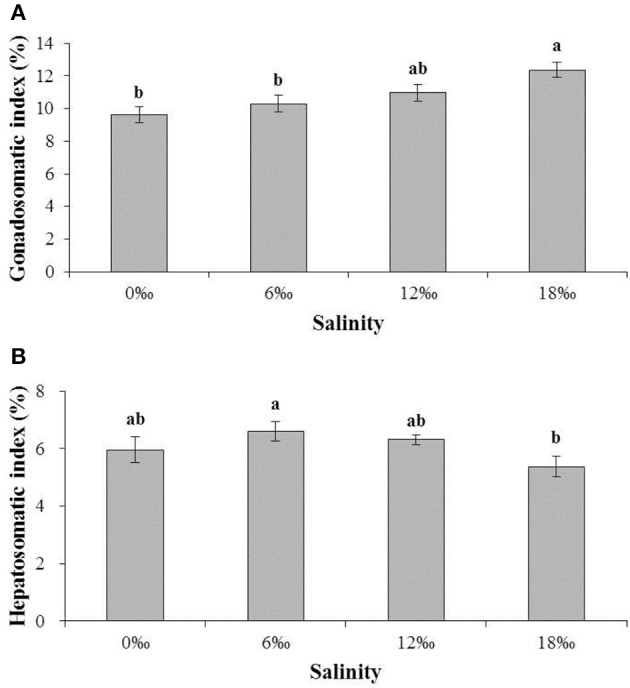
The gonadsomatic index (GSI) (%) and hepatosomatic index (HSI) (%) (± SE) of the female *E. sinensis* subjected to different salinities for 40 days. **(A)**: GSI; **(B)**: HSI. Different letters on the tops of the bars indicated significant differences.

The gonadosomatic index (GSI) of female *E. sinensis* showed a clear increasing trend with increasing salinity (Figure 2A) and the crabs from the highest salinity 18 ‰ treatment had significant higher GSI than other treatments (*P* < 0.05) except the 12‰ treatment. No significant difference was detected among the other three lower salinity treatments (*P* > 0.05).

The hepatosomatic index (HSI) showed a pattern of initially increased with salinity from 0 and 6 ‰, but subsequently decreased as salinity increased further to 18‰, resulting in the highest and the lowest HSI obtained from the 6 and 18 ‰ treatments, respectively, and a significant difference between the two treatments (*P* < 0.05, Figure 2B).

**Figure 3:**
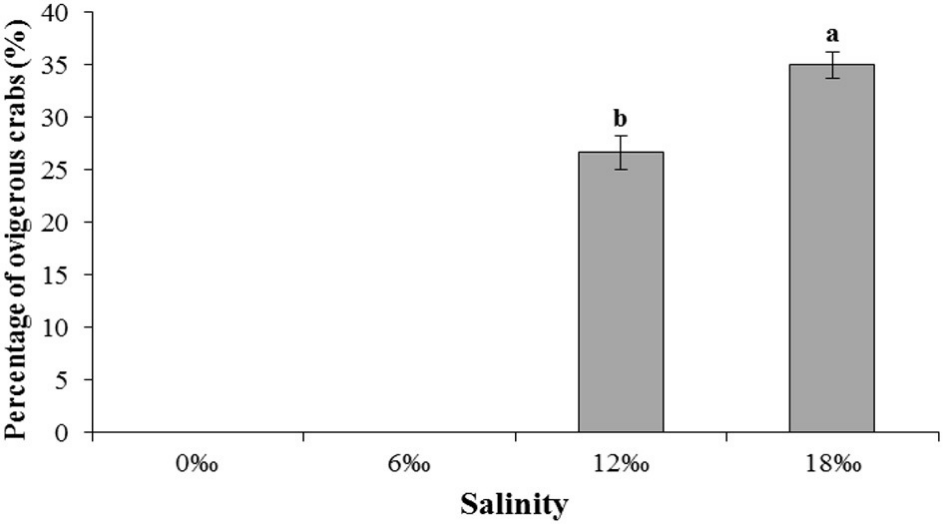
The percentage (%) (± SE) of vigorous female *E. sinensis* subjected to different salinities over 40 days. Different letters on the tops of the bars indicated significant differences.

Interestingly, egg carrying female crab was found without copulation in both the 12 and 18‰ treatments. By the end of the experiment on the day 40, the numbers of such ovigerous females in both treatments were substantial although the 18‰ treatment had significantly higher percentage (35.0%) than that of the 12‰ treatment (26.7%) (*P* < 0.05, Figure 3). No ovigerous crabs were found throughout the experiment in the other two lower salinity treatments (Figure 3). Microscopic examination of the spermathecaes of the ovigerous female crabs found no spermatophore within, confirming that the egg carrying female crabs in this study were indeed not copulated. Additionally, it was observed that all unfertilized eggs initially attached to ovigerous crabs failed to develop further and eventually dropped off within 2–3 days.

### Hemolymph osmolality, ionic concentrations, and free amino acid composition

**Figure 4:**
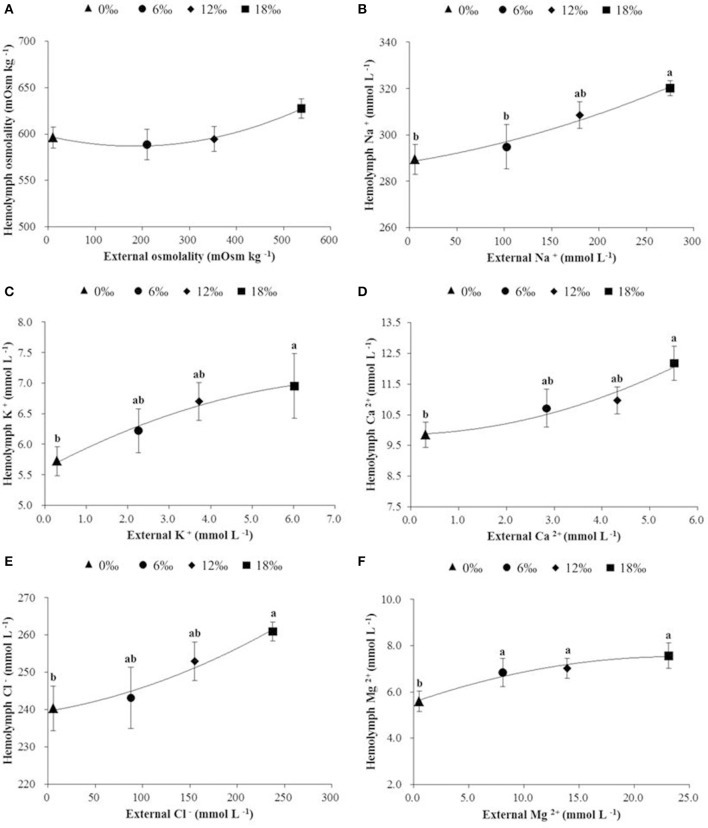
The haemolymph osmolality (mOsm kg ^−1^) (± SE) **(A)**, and concentrations (m mol L^−1^) of major ions (**B**: Na^+^, **C**: K^+^, **D**: Ca^2+^, **E**: Cl^−^, and **F**: Mg^2+^) in haemolymph of the female *E. sinensis* subjected to different salinities. Different letters indicated significant differences.

Hemolymph osmolality showed a trend of decrease initially (a minimum detected at 6‰) but subsequently increased as salinity increased from 0 to 18‰, however no significant difference was detected among all treatments (*P* < 0.05, Figure 4A). Of five major ions (Na^+^, K^+^, Ca^2+^, Cl^−^, and Mg ^2+^) measured, all showed an overall increasing trend with increasing salinity (*P* < 0.05), and except Mg ^2+^, the concentrations of the other four ions were always higher in the hemolymph than in the external media (Figures 4B–F).

Table 2 shows results of hemolymph free amino acid analysis. The total free amino acids (TFAAs) increased sharply with the increase of salinity from 0‰ (2500.60 ± 101.98 nmol mL^−1^) to 6‰ (3238.10 ± 41.70 nmol mL^−1^) but subsequently decreased as salinity further increased to 12‰ (2686.50 ± 116.63 nmol mL^−1^) and 18‰ (2594.30 ± 145.71 nmol mL^−1^). This resulted in 6‰ salinity treatment had significant higher TFAAs (*P* < 0.05) as well as the highest contents of major amino acids, including asparagine, glutamine, proline, alanine, citrulline, valine, methionine, isoleucine, leucine, phenylalanine, β-alanine, lysine, histidine and arginine. The most important contributors to TFAAs (>100 nmol mL^−1^), listed in the following on sequence of their relatively important, are: proline, alanine, taurine, arginine, glycine, and glutamine. Among these FAAs, proline, alanine, arginine and glutamine were the highest at 6‰ and significantly higher than other salinity treatments except alanine (*P* < 0.05). The alanine and glycine were not significantly different among all treatments while taurine increased with salinity to reach a peak at 12‰ but decreased at 18‰ (*P* < 0.05) (Table 2).

**Table 2:** Free amino acids composition (nmol mL^−1^) in hemolymph of female *E. sinensis* subjected to different salinities for 40 days.

**Free amino acids**	**Salinity**			
	**0‰**	**6‰**	**12‰**	**18‰**
Taurine	322.83 ± 24.85^b^	390.59 ± 20.49^b^	544.68 ± 32.73^a^	355.22 ± 27.09^b^
Aspartic acid	11.06 ± 1.18^c^	11.42 ± 0.62^c^	14.95 ± 1.41^b^	20.44 ± 0.89^a^
Threonine	47.12 ± 4.96^ab^	52.21 ± 4.92^a^	38.45 ± 3.60^b^	57.73 ± 1.73^a^
Serine	66.61 ± 6.27	65.53 ± 6.46	52.93 ± 2.69	66.43 ± 3.63
Asparagine	26.76 ± 2.28^c^	48.93 ± 3.59^a^	17.26 ± 1.91^d^	38.82 ± 3.02^b^
Glutamic acid	36.26 ± 1.69^b^	46.29 ± 4.72^b^	72.32 ± 3.11^a^	65.24 ± 7.75^a^
Glutamine	108.21 ± 9.13^c^	181.98 ± 9.68^a^	67.31 ± 5.51^d^	139.58 ± 7.24^b^
α-ABA	7.10 ± 0.49	9.23 ± 0.93	9.96 ± 1.21	7.59 ± 0.90
Proline	732.33 ± 55.91^b^	1007.50 ± 50.26^a^	714.47 ± 46.02^b^	710.21 ± 41.95^b^
Glycine	279.60 ± 28.66	246.19 ± 9.08	276.68 ± 15.51	246.71 ± 21.81
Alanine	440.55 ± 38.41	595.31 ± 43.40	493.92 ± 41.11	492.99 ± 72.60
Citrulline	3.39 ± 0.35^c^	6.52 ± 0.78^a^	5.39 ± 0.48^ab^	3.83 ± 0.32^bc^
α-AAA	4.54 ± 0.57	4.28 ± 0.25	4.23 ± 0.93	3.66 ± 0.17
Valine	32.88 ± 2.95^bc^	49.54 ± 3.56^a^	25.31 ± 2.88^c^	38.02 ± 3.47^b^
Methionine	4.28 ± 0.14^b^	4.98 ± 0.36^a^	0.87 ± 0.07^d^	3.11 ± 0.23^c^
Cystathionine	3.57 ± 0.70^a^	2.01 ± 0.29^b^	1.53 ± 0.19^b^	1.79 ± 0.20^b^
Isoleucine	19.75 ± 1.35^b^	25.39 ± 2.11^a^	14.46 ± 1.47^b^	18.14 ± 1.08^ab^
Leucine	27.23 ± 2.16^b^	37.98 ± 4.01^a^	21.32 ± 2.84^b^	26.61 ± 1.17^b^
Tyrosine	1.51 ± 0.23	1.40 ± 0.05	1.40 ± 0.09	1.62 ± 0.07
Phenylalanine	15.99 ± 1.28^b^	23.42 ± 0.52^a^	10.49 ± 0.89^c^	16.13 ± 1.01^b^
ß-Alanine	7.18 ± 0.37^b^	28.77 ± 3.71^a^	21.74 ± 0.37^a^	24.88 ± 2.28^a^
ß-AiBA	1.44 ± 0.16^b^	1.77 ± 0.20^b^	2.49 ± 0.11^a^	2.00 ± 0.29^ab^
Tryptophan	2.25 ± 0.26^b^	1.98 ± 0.17^b^	1.00 ± 0.07^c^	3.77 ± 0.52^a^
Ornithine	14.87 ± 1.68	16.33 ± 1.48	13.26 ± 0.82	17.17 ± 1.79
Lysine	42.14 ± 4.71^a^	44.78 ± 3.07^a^	23.58 ± 1.44^b^	29.10 ± 2.91^b^
Histidine	30.03 ± 1.79^b^	38.86 ± 3.32^a^	24.26 ± 2.11^b^	28.35 ± 2.22^b^
Arginine	232.16 ± 12.66^b^	292.50 ± 28.25^a^	211.17 ± 13.48^b^	174.81 ± 13.83^b^
TFAAs	2500.60 ± 101.98^b^	3238.10 ± 41.70^a^	2686.50 ± 116.63^b^	2594.30 ± 145.71^b^

*Data are presented as mean ± SE. Values within the same row with different letters mean significant difference (P < 0.05). Free amino acid contents < 1 nmol mL^−1^ are not listed in this table. α-ABA: α- amino-n-butyric acid; α-AAA: α-aminoadipicacid; ß-AiBA: β-aminoisobutyric acid; TFAAs: total free amino acids*.

### Gill Na^+^/K^+^-ATPase and its mRNA expression

**Figure 5:**
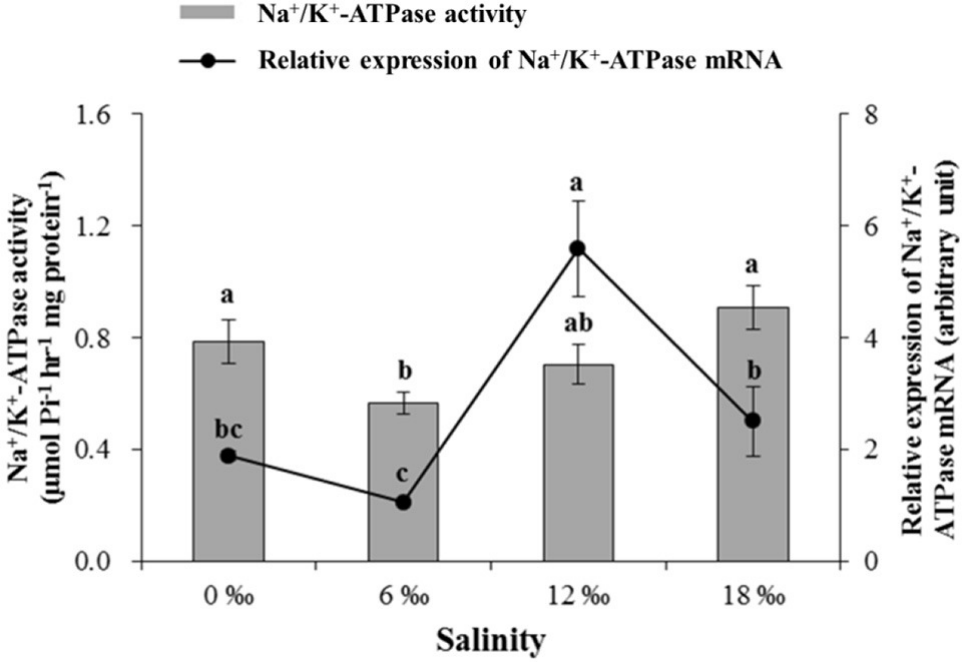
The gill Na^+^/K^+^-ATPase activity (μmol Pi h^−1^ mg protein^−1^) and its mRNA expression level (± SE) in the posterior gills of the female *E. sinensis* subjected to different salinities for 40 days.

The activity of Na^+^/K^+^-ATPase in the posterior gills decreased significantly with salinity increased from 0 to 6 ‰ but started to increase as salinity further increased to 12 and 18‰, resulting in the lowest Na^+^/K^+^-ATPase activity being recorded at 6‰, which was significantly lower than all other salinity treatments except the 12‰ treatment (*P* < 0.05, Figure 5). The expression level of Na^+^/K^+^ -ATPase mRNA in the posterior gills showed a fluctuating pattern with the highest and the lowest level detected at 12 and 6‰, respectively (*P* < 0.05) (Figure 5).

### Gill fatty acids profile

The fatty acids profile in the anterior gills is shown in Table 3. The highest percentages of major saturated fatty acids (SFAs) and total saturated fatty acids (∑SFA) were detected at 12‰ (*P* < 0.05). For monounsaturated fatty acids (MUFAs), the levels of C18:1n7, C20:1n7 and total monounsaturated fatty acids (∑MUFA) firstly increased significantly with salinity to reach a peak at 6‰ but then decreased significantly with further increase in salinity (*P* < 0.05). As to polyunsaturated fatty acids (PUFAs), the C18:2n6, C20:3n6, C20:4n6 and total n-6 polyunsaturated fatty acids (∑n-6 PUFA) showed a fluctuating pattern with the highest values detected at 18‰ (*P* < 0.05). On the other hand, the C18:3n3 increased significantly with salinity initially to reach a peak at 12 ‰ before it decreased significantly at 18‰ (*P* < 0.05). The C20:2n6, ∑PUFA and total highly unsaturated fatty acids (∑HUFA) firstly showed a decreasing trend with increasing salinity with a minimum detected for 12‰ treatment before it increased as salinity increased to 18‰ (*P* < 0.05). Meanwhile, the highest C22:6n3 was detected at 6‰ while the ∑n-3 PUFA and n-3/n-6 PUFA ratio showed a decreasing trend with increasing salinity (*P* < 0.05).

**Table 3:** Fatty acids profile (% of total fatty acids) in the anterior gills of female *E. sinensis* subjected to different salinities for 40 days.

**Fatty acids**	**Salinity**			
	**0‰**	**6‰**	**12‰**	**18‰**
C14:0	0.30 ± 0.01^bc^	0.32 ± 0.01^b^	0.44 ± 0.02^a^	0.28 ± 0.01^c^
C15:0	0.20 ± 0.00^bc^	0.21 ± 0.01^b^	0.27 ± 0.02^a^	0.17 ± 0.00^c^
C16:0	11.22 ± 0.06^c^	12.59 ± 0.29^b^	15.49 ± 0.54^a^	13.04 ± 0.27^b^
C17:0	0.35 ± 0.00^b^	0.31 ± 0.01^b^	0.44 ± 0.05^a^	0.28 ± 0.01^b^
C18:0	7.14 ± 0.13^bc^	6.71 ± 0.15^c^	8.58 ± 0.13^a^	7.45 ± 0.15^b^
C20:0	0.72 ± 0.00^b^	0.74 ± 0.02^b^	0.92 ± 0.04^a^	0.74 ± 0.02^b^
C22:0	0.73 ± 0.01^b^	0.84 ± 0.02^b^	1.10 ± 0.12^a^	0.86 ± 0.02^b^
∑SFA	20.67 ± 0.20^c^	21.74 ± 0.51^bc^	27.24 ± 0.33^a^	22.81 ± 0.47^b^
C16:1n7	2.91 ± 0.02^b^	3.64 ± 0.08^a^	3.85 ± 0.18^a^	3.16 ± 0.07^b^
C18:1n9	18.99 ± 0.22	19.93 ± 0.46	19.55 ± 0.10	19.47 ± 0.40
C18:1n7	3.12 ± 0.04^b^	3.67 ± 0.08^a^	3.23 ± 0.03^b^	3.09 ± 0.07^b^
C20:1n7	0.81 ± 0.01^b^	0.87 ± 0.02^a^	0.72 ± 0.02^c^	0.82 ± 0.02^ab^
∑MUFA	25.83 ± 0.31^b^	28.13 ± 0.65^a^	27.35 ± 0.28^ab^	26.55 ± 0.55^ab^
C18:2n6	7.02 ± 0.03^c^	7.52 ± 0.17^ab^	7.20 ± 0.09^bc^	7.93 ± 0.16^a^
C18:3n3	0.38 ± 0.00^b^	0.44 ± 0.01^a^	0.45 ± 0.01^a^	0.40 ± 0.01^b^
C20:2n6	2.30 ± 0.04^a^	2.09 ± 0.05^b^	1.79 ± 0.09^c^	2.39 ± 0.05^a^
C20:3n6	0.17 ± 0.01^b^	0.20 ± 0.00^ab^	0.08 ± 0.03^c^	0.21 ± 0.01^a^
C20:4n6	18.20 ± 0.38^b^	18.58 ± 0.43^b^	16.72 ± 0.25^c^	20.48 ± 0.42^a^
C20:5n3	9.27 ± 0.19^a^	8.22 ± 0.19^b^	7.56 ± 0.14^c^	7.07 ± 0.15^c^
C22:6n3	4.78 ± 0.10^ab^	5.00 ± 0.11^a^	4.17 ± 0.14^c^	4.45 ± 0.09^bc^
∑PUFA	42.13 ± 0.75^a^	42.04 ± 0.97^a^	37.95 ± 0.56^b^	42.92 ± 0.88^a^
n-3PUFA	14.43 ± 0.29^a^	13.65 ± 0.32^a^	12.18 ± 0.28^b^	11.92 ± 0.25^b^
n-6PUFA	27.69 ± 0.46^b^	28.39 ± 0.66^b^	25.77 ± 0.28^c^	31.01 ± 0.64^a^
n-3/n-6	0.52 ± 0.00^a^	0.48 ± 0.00^b^	0.47 ± 0.01^c^	0.38 ± 0.00^d^
∑HUFA	32.42 ± 0.68^a^	31.99 ± 0.74^a^	28.50 ± 0.55^b^	32.21 ± 0.66^a^

*Data are presented as mean ± SE. Values within the same row with different letters indicate significant difference (P < 0.05). ∑SFA: total saturated fatty acid; ∑MUFA: total monounsaturated fatty acid; ∑PUFA: total polyunsaturated fatty acid; ∑HUFA: total highly unsaturated fatty acid*.

In the case of posterior gills, the percentages of most major SFAs increased significantly with salinity to reach a peak at 12‰ (*P* < 0.05) before decreased at 18‰, on the other hand, the highest C16:0 and ∑SFA was detected at 6‰ treatment (*P* < 0.05) (Table 4). For MUFAs, the 6 ‰ treatment resulted in the highest levels of C16:1n7, C18:1n9, C18:1n7 and ∑MUFA (*P* < 0.05). Regarding PUFAs, the percentage of C18:2n6 showed a fluctuating pattern with the highest value detected at 6‰, while the C18:3n3 increased significantly with salinity to reach a peak at 12‰ before decreased significantly at 18‰ (*P* < 0.05). The 0‰ treatment resulted in the highest percentages of C20:2n6, C20:5n3, n-3PUFA as well as n-3/n-6 ratio, while the C20:3n6 and C20:4n6 increased significantly with increasing salinity (*P* < 0.05). The C22:6n3, ∑PUFA and∑HUFA showed a trend of initially decreased (a minimum detected at 6‰) but then increased with increasing salinity, while ∑n-6 PUFA showed an overall increasing trend with increasing salinity (*P* < 0.05) (Table 4).

**Table 4:** Fatty acids profile (% of total fatty acids) in the posterior gills of female *E. sinensis* subjected to different salinities for 40 days.

**Fatty acids**	**Salinity**			
	**0‰**	**6‰**	**12‰**	**18‰**
C14:0	0.18 ± 0.00^b^	0.26 ± 0.01^a^	0.27 ± 0.01^a^	0.27 ± 0.01^a^
C15:0	0.15 ± 0.00^b^	0.17 ± 0.01^a^	0.18 ± 0.00^a^	0.17 ± 0.00^a^
C16:0	10.57 ± 0.24^d^	14.03 ± 0.33^a^	12.43 ± 0.22^b^	11.52 ± 0.27^c^
C17:0	0.31 ± 0.01^b^	0.34 ± 0.01^b^	0.39 ± 0.03^a^	0.32 ± 0.01^b^
C18:0	6.80 ± 0.16^b^	7.58 ± 0.18^a^	7.71 ± 0.06^a^	7.53 ± 0.18^a^
C20:0	0.71 ± 0.02^b^	0.73 ± 0.02^b^	0.82 ± 0.01^a^	0.81 ± 0.02^a^
C22:0	0.75 ± 0.02^b^	0.73 ± 0.02^b^	0.93 ± 0.00^a^	0.92 ± 0.02^a^
∑SFA	19.47 ± 0.45^c^	23.84 ± 0.55^a^	22.73 ± 0.26^ab^	21.55 ± 0.50^b^
C16:1n7	2.50 ± 0.06^d^	3.75 ± 0.09^a^	3.07 ± 0.02^b^	2.83 ± 0.07^c^
C18:1n9	17.92 ± 0.41^c^	20.76 ± 0.48^a^	19.38 ± 0.00^b^	19.68 ± 0.46^ab^
C18:1n7	2.88 ± 0.07^c^	3.36 ± 0.08^a^	3.09 ± 0.01^b^	3.03 ± 0.07^bc^
C20:1n7	0.74 ± 0.02	0.78 ± 0.02	0.75 ± 0.00	0.78 ± 0.02
∑MUFA	24.04 ± 0.56^c^	28.65 ± 0.66^a^	26.29 ± 0.03^b^	26.32 ± 0.61^b^
C18:2n6	7.89 ± 0.18^b^	8.86 ± 0.21^a^	7.73 ± 0.04^b^	8.23 ± 0.19^b^
C18:3n3	0.45 ± 0.01^b^	0.48 ± 0.01^ab^	0.50 ± 0.01^a^	0.45 ± 0.01^b^
C20:2n6	3.00 ± 0.07^a^	2.55 ± 0.06^b^	2.58 ± 0.01^b^	2.49 ± 0.06^b^
C20:3n6	0.15 ± 0.00^c^	0.18 ± 0.00^b^	0.19 ± 0.00^b^	0.25 ± 0.01^a^
C20:4n6	14.80 ± 0.34^c^	15.63 ± 0.36^bc^	16.67 ± 0.04^b^	18.68 ± 0.43^a^
C20:5n3	11.69 ± 0.29^a^	8.85 ± 0.20^c^	9.89 ± 0.04^b^	8.91 ± 0.21^c^
C22:6n3	4.35 ± 0.10^c^	4.01 ± 0.09^d^	4.68 ± 0.02^b^	5.02 ± 0.12^a^
∑PUFA	42.34 ± 0.98^ab^	40.55 ± 0.93^b^	42.24 ± 0.01^ab^	44.03 ± 1.02^a^
n-3PUFA	16.50 ± 0.38^a^	13.34 ± 0.31^c^	15.08 ± 0.01^b^	14.38 ± 0.33^b^
n-6PUFA	25.84 ± 0.60^b^	27.21 ± 0.63^b^	27.16 ± 0.00^b^	29.65 ± 0.69^a^
n-3/n-6	0.64 ± 0.00^a^	0.49 ± 0.00^c^	0.56 ± 0.00^b^	0.49 ± 0.00^c^
∑HUFA	30.99 ± 0.72^a^	28.67 ± 0.66^b^	31.43 ± 0.06^a^	32.86 ± 0.76^a^

*Data are presented as mean ± SE. Values within the same row with different letters indicate significant difference (P < 0.05). ∑SFA: total saturated fatty acid; ∑MUFA: total monounsaturated fatty acid; ∑PUFA: total polyunsaturated fatty acid; ∑HUFA: total highly unsaturated fatty acid*.

### Hemolymph and hepatopancreas metabolism indices

Various metabolism indices measured in hemolymph showed that GLU generally decreased with increasing salinity and was significantly lower at 12 and 18‰ while the highest content of TC was detected at 12‰ (*P* < 0.05, Table 5). No significant differences were detected among four salinity treatments for TG, HDL-C, LDL-C, UA and urea in hemolymph (*P* > 0.05). The levels of SOD, MDA and γ-GT showed a fluctuating pattern, but the highest and the lowest contents of all these indices were consistently found at 0 and 6‰, respectively. On the other hand, the highest activities of ALP and ACP were detected at 18‰ (*P* < 0.05).

**Table 5:** Hemolymph metabolism indices of female *E. sinensis* subjected to different salinities for 40 days.

**Indices**	**Salinity**			
	**0‰**	**6‰**	**12‰**	**18‰**
GLU (mmol L^−1^)	4.98 ± 0.26^a^	4.77 ± 0.25^a^	3.02 ± 0.20^b^	3.10 ± 0.26^b^
TC (mmol L^−1^)	0.48 ± 0.06^ab^	0.41 ± 0.05^b^	0.63 ± 0.08^a^	0.57 ± 0.06^ab^
TG (mmol L^−1^)	0.20 ± 0.01	0.20 ± 0.02	0.24 ± 0.02	0.24 ± 0.04
HDL-C (mmol L^−1^)	0.22 ± 0.03	0.23 ± 0.02	0.24 ± 0.02	0.26 ± 0.02
LDL-C (mmol L^−1^)	0.16 ± 0.02	0.16 ± 0.03	0.19 ± 0.02	0.14 ± 0.01
UA (mmol L^−1^)	43.86 ± 5.14	40.77 ± 7.42	51.18 ± 6.04	41.65 ± 5.55
Urea (μmol L^−1^)	2.75 ± 0.20	2.57 ± 0.18	2.80 ± 0.49	2.89 ± 0.36
SOD (U mL^−1^)	91.86 ± 1.12^a^	85.57 ± 0.88^b^	88.27 ± 1.80^ab^	90.57 ± 1.54^a^
MDA (nmol mL^−1^)	21.06 ± 1.62^a^	14.99 ± 1.22^b^	19.00 ± 1.91^ab^	17.12 ± 1.54^ab^
γ-GT (U L^−1^)	0.72 ± 0.06^a^	0.36 ± 0.04^b^	0.89 ± 0.14^a^	1.04 ± 0.23^a^
ALP (U L^−1^)	4.03 ± 0.25^b^	3.93 ± 0.33^b^	2.80 ± 0.04^c^	5.22 ± 0.40^a^
ACP (U 100 mL^−1^)	1.04 ± 0.08^b^	1.28 ± 0.13^b^	1.23 ± 0.16^b^	1.74 ± 0.16^a^

*Data are presented as mean ± SE. Values within the same row with different letters mean significant difference (P < 0.05). GLU: glucose; TC: total cholesterol; TG: triglyceride; HDL-C: high-density lipoprotein cholesterol; LDL-C: low-density lipoprotein cholesterol; UA: uric acid; SOD: superoxide dismutase; MDA: malonaldehyde; γ-GT: γ-glutamyltransferase; ALP: alkaline phosphatase; and ACP: acid phosphatase*.

Table 6 showed metabolism indices measured in hepatopancreas of the female crabs from different salinity treatments. The GLU content as well as the activities of ALP and ACP showed a same pattern of firstly decreased with salinity with the lowest values recorded at 6‰ but increased subsequently with increasing salinity (*P* < 0.05). No significant differences were detected for the contents of UA and MDA as well as the activity of SOD among all treatments (*P* > 0.05). The activity of γ-GT decreased consistently with increasing salinity (*P* < 0.05) while urea showed a fluctuating pattern with the highest level detected at 18‰ (*P* < 0.05).

**Table 6:** Hepatopancreas metabolism indices of female *E. sinensis* subjected to different salinities for 40 days.

**Indices**	**Salinity**			
	**0‰**	**6‰**	**12‰**	**18‰**
GLU (mmol g^−1^ tissue)	19.41 ± 1.68^ab^	17.18 ± 1.31^b^	19.17 ± 2.39^ab^	22.24 ± 0.76^a^
UA (mmol g^−1^ protein)	2.79 ± 0.45	2.25 ± 0.31	2.86 ± 0.30	2.60 ± 0.55
Urea (μmol g^−1^ protein)	0.30 ± 0.03^ab^	0.30 ± 0.03^ab^	0.27 ± 0.03^b^	0.40 ± 0.02^a^
SOD (U g^−1^ protein)	28.53 ± 1.32	23.57 ± 3.51	26.96 ± 2.01	23.37 ± 1.83
MDA (nmol g^−1^ protein)	2.38 ± 0.25	2.21 ± 0.25	3.05 ± 0.37	2.89 ± 0.43
γ-GT (U g^−1^ protein)	8.24 ± 1.06^a^	5.94 ± 1.17^b^	3.94 ± 0.33^bc^	3.25 ± 0.30^c^
ALP (U g^−1^ protein)	9.59 ± 1.07^a^	5.58 ± 0.40^b^	6.37 ± 0.81^b^	7.90 ± 0.51^ab^
ACP (U 100 g^−1^ protein)	3.18 ± 0.32^ab^	2.39 ± 0.10^b^	2.63 ± 0.32^b^	3.73 ± 0.27^a^

*Data are presented as mean ± SE. Values within the same row with different letters mean significant difference (P < 0.05). GLU: glucose; UA: uric acid; SOD: superoxide dismutase; MDA: malonaldehyde; γ-GT: γ-glutamyltransferase; ALP: alkaline phosphatase; and ACP: acid phosphatase*.

## Discussion

Salinity is a key environmental parameter that affects the survival, gonadal development and reproduction of the catadromous *E. sinensis* (Zhang et al., 2001; Cheng et al., 2008; Wu et al., 2013). In the current study, the female *E. sinensis* subjected to four salinity conditions ranging from 0 to 18‰ showed mortality mainly after day 20, and higher mortalities were found at higher salinity treatments (12 and18‰). This may be explained by the observation that under higher salinities, female *E. sinensis* generally showed substantial higher level of activity (Zhuang et al., 2012), which may lead to higher incidences of aggressive encounters, injuries and cannibalism, particularly when only females were used and kept together in a confined area such as in the current study. In fact, the GSI of the female *E. sinensis* from the highest salinity treatment (18‰) was significantly higher than those form lower salinity treatments (0 and 6‰), which suggests that the higher salinity condition in the former accelerated ovarian development of female *E. sinensis*. Similar results were found for male *E. sinensis* form a previous study by the authors (Long et al., 2017b). Such advanced gonad development under higher salinities probably triggered increased activities of the females in searching of mating males. The accelerated ovarian development under higher salinities might be explained by: (1) the osmolality of higher salinity water is closer to that of the hemolymph of the female *E. sinensis*, the crabs hence could have reduced energy expenditure on osmoregulation (Lee and Chen, 2003; Jia et al., 2012) while channeled more nutrients/energy to ovarian development (Wu et al., 2013); (2) elevating salinity increased hemolymph ion concentrations, which included calcium while calcium is known to have the function of promoting synthesis and absorption of vitellogenin in crustaceans (Quinito et al., 1989; Wei et al., 2005, 2007); (3) higher salinity brackish water may lead to improved hemolymph estrogen level, such as 17β-estradiol (E2), which activated/enhanced ovarian development (Wu and Jiang, 2001; Wei et al., 2007; Wu et al., 2013). On the other hand, HSI of the female *E. sinensis* showed an overall decreasing trend with increasing salinity, which is probably related to more hepatopancreas reserve being transferred to the ovaries under higher salinity conditions to facilitate ovarian development and maturation (Cheng et al., 1998; Teng et al., 2008).

It is worth noting that during the current experiment, egg carrying females were found in the two higher salinity treatments (12 and 18 ‰) despite no copulation had occurred as all crabs used were females. The observation that female crabs spawned without copulation was confirmed by checking spermathecaes of the ovigerous females under a microscope and no spermatophore was found in any of them. While spawning without copulation has not been reported previously for *E. sinensis*, in the giant freshwater prawn *Macrobrachium rosenbergii*, unmated ripe females have been reported to lay eggs within 24 h of pre-mating molt, but the unfertilised eggs drop off in 2–3 days (New et al., 2010); similar situation was also observed in the harlequin crab *Lissocarcenus laevis* (C. Zeng, personal observation). Similarly, in this study, all unfertilized eggs that initially attached to ovigerous crabs failed to develop further and eventually dropped off within a few days. It is likely that the favorable higher salinity conditions (12 and 18‰) triggered rapid ovarian development that became irreversible, eventually led to unfertilized eggs being extruded by the females despite no copulation had happened. On the other hand, for those females kept in freshwater or low salinity conditions, oocyte development was much slower and probably were later re-absorbed as the external condition remained unfavorable.

Past studies reported clear differences in salinity of respective distribution areas of male and female *E. sinensis* in estuaries when they first arrived there for reproduction (Lai, 1994; Zhang and Li, 2002; Bentley, 2011). As such, there are likely gender differences in physiological responses to salinity changes, such as osmoregulation and metabolism. Gills play an important role in osmotic and ionic regulation in crustaceans (Mendonça et al., 2007; Romano and Zeng, 2010). Previous studies have demonstrated that the osmotic and ionic regulation in the posterior gills is dependent on ionic transport enzymes (e.g., Na^+^/K^+^-ATPase) and proteins (Kaplan, 2002; Henry et al., 2003). In this study, the activity of Na^+^/K^+^-ATPase in the posterior gills decreased significantly as salinity increased from 0 at 6‰ but increased with further increase in salinity with a significant higher activity recorded at 18‰. Such results may be related to the changes in free amino acid contents in the hemolymph under different salinities. In addition to ions, free amino acids (FAAs) are also known to contribute to hemolymph osmolality and play an important role in osmoregulation in crustaceans (Kempf and Bremer, 1998; Huong et al., 2001). Our results showed that the most free amino acids and total amino acid (TFAAs) in hemolymph of the female crabs increased significantly to the highest levels as salinity increased from 0 to 6‰, it subsequently decreased as salinity increased further to 18‰. Such a trend differs from Na^+^/K^+^-ATPase activity in posterior gills which showed the lowest level at 6‰. The significantly higher TFAAs coincidental with the lowest gill Na^+^/K^+^-ATPase activity detected at 6‰ suggests that compared to other salinity conditions, FFAs likely played a more significant role in osmoregulation at 6‰, which effectively reduced the need for ion transportation fueled by Na^+^/K^+^-ATPase. As the result, Na^+^/K^+^-ATPase activity level and energy expenditure by the female crabs on osmoregulation was reduced. However, it is worth noting that a previous similar study by the author on male *E. sinensis* showed very different results, that the activity of Na^+^/K^+^-ATPase in the posterior decreased significantly with increasing water salinity (Long et al., 2017b). A possible explanation for such a gender difference in Na^+^/K^+^-ATPase activity could be that for the female crabs subjected to higher salinities (12 and 18‰), the rapid ovarian and oocyte development requires higher levels of certain irons, such as Ca^2+^, which is known to play an important role in regulating vitellogenesis in crustaceans (Paulus and Laufer, 1987; Quinito et al., 1989). To achieve that, Na^+^/K^+^-ATPase activity in the posterior gills of the female crabs subjected to higher salinities had to maintain at higher level than that of the males under similar salinity conditions.

Gill fatty acid profile can also reflect the physiological responses of crabs to environmental salinity changes (Lucu et al., 2008; Romano et al., 2014). The female *E. sinensis* subjected to higher salinity conditions were found to have higher total saturated fatty acids and total monounsaturated fatty acids in their anterior and posterior gills, similar results was reported for the amphipod *Gammarus duebeni* (Morris et al., 1982). This may be related to these fatty acids are the major energy source, hence their higher contents in the gills were linked to high energy requirements for respiration and osmoregulation in hypo-osmotic environment (Welcomme and Devos, 1991). Meanwhile, total polyunsaturated fatty acids and total highly unsaturated fatty acids in the posterior gills showed a trend of firstly decreased with increasing salinity (minimum detected at 6‰) but subsequently increased significantly as salinity increased further, which was consistent with the trend of Na^+^/K^+^-ATPase activity changes with salinity detected in the posterior gills. Such a pattern suggests that the membrane fluidity and Na^+^/K^+^ -ATPase activity in the posterior gills of female *E. sinensis* may be modulated by its fatty acid profile (Palacios et al., 2004).

The metabolism indices generally reflect the physiological status of *E. sinensis* exposed to different salinities (Jia et al., 2012; Feng et al., 2013). In this study, the contents of hemolymph glucose (GLU) in the female crabs subjected to higher salinity treatments of 12 and 18‰ were significantly lower than those from the low salinity treatments (0 and 6‰). GLU is a major energy source for osmoregulation (Lorenzon et al., 1997; Al-Azhary et al., 2008); since the osmolality of higher salinity waters (12 and 18‰) were closer to the osmolality in the hemolymph when compared to the low salinities (0 and 6‰); fewer energy is expected to be required for osmoregulation by the crabs kept in salinity 12 and 18‰ (Jia et al., 2012), hence less GLU was produced. Meanwhile, cholesterol is not only a precursor of various hormones that regulate ovarian development and molt, but also an important component of cell membranes in crustaceans (Quackenbush, 1986; Cheng et al., 2000; Wei et al., 2005). In the current study, hemolymph total cholesterol level was the highest at 12 and 18‰, which may relate to accelerated ovarian development of the crabs under higher salinity conditions (Wu et al., 2013).

Urea is a metabolite of proteins, and its content generally reflects level of protein metabolism (Chen and Lin, 1995; Chang et al., 2007). In this study, the 18‰ treatment resulted in the highest content of urea in the hepatopancreas, a similar result was reported for the Kuruma shrimp *Marsupenaeus japonicus* (Lee and Chen, 2003). Previous studies have shown that ammonia nitrogen could be converted to lower toxic urea, amino acids and other nitrogen compounds with elevated external salinities by crustaceans (Cheng and Chen, 1998; Lee and Chen, 2003), which may imply lower ammonia excretion of the female *E. sinensis* in high salinity waters.

Superoxide dismutase (SOD) is an important antioxidant enzyme that can scavenge free radicals and prevents tissue damages in crustaceans (Li et al., 2008; Lu et al., 2011). The 6‰ salinity resulted in the lowest hemolymph SOD activity compared to other salinities, suggesting relatively lower level of free radicals in hemolymph of the female *E. sinensis* under the salinity (Chien et al., 2003; Wang et al., 2012). Meanwhile, malondialdehyde (MDA) is an indicator of lipid peroxidation, its content often reflects the levels of free radicals and cellular oxidation in tissues (Long et al., 2017a), both hemolymph and hepatopancrea MDA of the crabs were the lowest at 6‰ as compared to other salinities, again indicating lower peroxide stress in tissues at the salinity.

## Conclusions

The present study demonstrated that brackish water accelerated ovarian development of female *E. sinensis*, in particular 12 and 18 ‰ salinities also led to unusual phenomenon of occurrence of ovigerous crabs without copulation, as well as generally lower levels of metabolism.

## Ethics statement

This study was conducted to investigate the effects of water salinity on gonadal development, osmoregulation and metabolism of adult female *E. sinensis*. The gonadal development situation of female crabs were checked via dissection, while the osmolality, major ions and free amino acids of hemolymph, gill Na^+^/K^+^-ATPase activity and its mRNA expression levels and fatty acids profile as well as the metabolism indices in hemolymph and hepatpancreas was analyzed with biochemical and molecular biology experimental methods. Prior to the sampling, all crabs will be treated with cold shock method to minimize suffering.

## Author contributions

XL, XW, CZ, and YC contributed to the conception and design of the work; XL, XW, and LZ contributed to data acquisition, analysis, and interpretation; XL, XW, CZ, HY, and YC drafted the work and revised it critically; XL, XW, LZ, HY, YC, and CZ agree to be accountable for all aspects of the work and ensure that questions related to the accuracy and integrity of all parts of the work are appropriately investigated and resolved.

### Conflict of interest statement

The authors declare that the research was conducted in the absence of any commercial or financial relationships that could be construed as a potential conflict of interest.
